# The more “mental miles” traveled during daydreaming task, the greater one's susceptibility to false fame: further evidence in support of a contextual change account of the false fame effect

**DOI:** 10.3389/fcogn.2026.1941362

**Published:** 2026-10-06

**Authors:** Dorothy R. Buchli

**Affiliations:** Department of Psychology, Mercer University, Macon, GA, United States

**Keywords:** autobiographical memories, context dependent memory, daydreaming, directed forgetting, false fame effect

## Abstract

The false fame effect occurs when previously encountered nonfamous names are later misidentified as famous because, although people no longer remember the circumstances in which the names were encountered, the names remain familiar and that familiarity is mistakenly attributed to fame. The present study examined whether shifting participants' internal context through autobiographical daydreaming would increase susceptibility to the false fame effect, and whether larger context shifts would produce greater memory distortions. Across two experiments, participants first studied a list of nonfamous names before completing a task designed to induce either a near or far shift in internal context. In Experiment 1, participants mentally reconstructed either their own home or their parents' home. In Experiment 2, they described either a recent domestic vacation or an international vacation. Control participants completed a reading task that did not encourage mental context change. Participants then studied a list of famous names and completed a fame judgment task. Across both experiments, participants who experienced a far context change made significantly more false fame judgments than those who experienced a near context change, who in turn made more false fame judgments than control participants. Moreover, across the near and far context-change conditions, the number of “mental miles” traveled during the daydreaming task was positively correlated with false fame judgments in both experiments. Thus, greater mental distance was associated with greater susceptibility to false fame. Signal detection analyses likewise revealed reduced discrimination between famous and nonfamous names following larger context shifts. Together, these findings demonstrate that greater internal contextual change increases susceptibility to the false fame effect, providing further support for a contextual change account.

## Introduction

A wealth of evidence suggests that many innocent individuals have been tried, convicted, and sent to prison for crimes they did not commit. These false convictions are primarily due to mistaken identification from an eyewitness (Wells et al., 2020). For instance, in a now famous case of misidentification, a ticket agent working at a train station was robbed and later identified a sailor as the perpetrator when asked to view a police lineup. However, the sailor was able to prove that he was not at the train station on the night of the incident. When the ticket agent was pressed to explain why he believed the sailor was the perpetrator, he said that the sailor looked familiar to him. This sense of familiarity however, derived from the fact that the sailor had been served by the ticket agent on several occasions before the crime was committed. Thus, the ticket agent incorrectly attributed the source of the familiarity he felt upon seeing the sailor again to the robbery and not their prior interactions (Goldstein, 2018; Ross et al., 1994).

Another well documented lapse of memory that results from misattribution of familiarity is the false fame effect. In the first experiment demonstrating this phenomenon, Jacoby et al. (1989) asked participants to read a series of nonfamous names aloud, such as *Sebastian Weisdorf* or *Valerie Marsh*, so the researchers could measure the speed of their pronunciation. Participants were then asked to choose, either immediately or 24 h later, which names were famous when presented with a list comprised of the same nonfamous names, new nonfamous names, and famous names (e.g., *Mario Andretti)*. When participants in the immediate test condition completed the fame judgment task, only about 12% of the old nonfamous names were erroneously selected as famous. This number rose to 16% however, in the delayed test condition. The authors posited that the greater proportion of false fame judgments in the delayed test condition reflected the fact that participants' sense of familiarity with the names was intact, but they were unable to recall the particular circumstances in which they previously encountered the names. That is, when there was no retention interval between the initial presentation of the nonfamous names and the fame judgment task, participants were easily able to distinguish between the nonfamous names they previously studied and the new famous names. However, when the fame judgment task was completed after a delay, recollection of the earlier learning episode was significantly impaired. While participants failed to remember having previously been exposed to the nonfamous names, the names continued to elicit a vague sense of familiarity, and this familiarity was incorrectly attributed to fame. Thus, judgments of false fame result from errors related to source memory. In a subsequent experiment, Jacoby et al. (1989) demonstrated that prompting participants to provide the source of the name greatly reduced the proportion of false fame judgments. Studies of aging and false fame demonstrate that older adults are more vulnerable to false fame effects than younger adults, primarily due to source monitoring difficulties that are largely confined to the domain of verbal memory (Dywan and Jacoby, 1990; Multhaup, 1995).

## Explaining the false fame effect: the contextual change hypothesis

Several follow-up studies provided converging evidence that false fame effects result from an intact sense of familiarity with the nonfamous names in the absence of conscious recollection of the earlier learning episode. Particularly relevant is research using the directed forgetting paradigm, in which participants are explicitly instructed to forget information they have recently encoded. Early demonstrations showed that instructions to forget could substantially reduce the influence of previously presented information on subsequent memory performance (Bjork, 1970, 1972; Geiselman et al., 1983). In the list-method version of this paradigm, participants typically study one list of items (List 1), receive an instruction either to forget or continue remembering that list, and then study a second list (List 2). Despite subsequently being asked to recall items from both lists, participants who receive the forget instruction generally recall fewer List 1 items than participants instructed to remember both lists, a phenomenon referred to as the *cost* of directed forgetting. In many circumstances, the forget instruction also improves memory for subsequently presented List 2 items, producing a *benefit* of directed forgetting (Bäuml, 2008; Bjork et al., 1998; MacLeod, 1998).

In a clever adaptation of the list-method directed forgetting paradigm, Bjork and Bjork (2003) demonstrated that these costs can extend beyond failures of explicit recall to errors in source attribution. Participants first studied a list of nonfamous names and were subsequently cued either to remember or forget that list before learning a second list consisting of famous names. After a brief distractor task, participants completed a fame judgment task containing old and new famous and nonfamous names. Participants instructed to remember the first list erroneously judged 36% of the previously studied nonfamous names to be famous, whereas participants instructed to forget the list made false fame judgments for 52% of those names. Bjork and Bjork (2003) attributed this increase in false fame to retrieval inhibition: the forget instruction impaired participants' ability to consciously recollect the initially presented nonfamous names without eliminating the familiarity produced by their earlier exposure. Consequently, when an old nonfamous name subsequently elicited familiarity, participants were more likely to misattribute that familiarity to fame.

An alternative account of list-method directed forgetting, however, proposes that at least some of the forgetting produced by a forget instruction reflects a change in mental context rather than inhibition of the memory representation itself (Sahakyan and Kelley, 2002). According to the contextual-change account, contextual features present during an event become associated with the information encoded during that event and can later function as retrieval cues (Estes, 1955; Howard and Kahana, 2002; Mensink and Raaijmakers, 1988). Participants instructed to remember both lists are likely to treat them as parts of the same continuous learning episode, allowing contextual elements associated with List 1 to remain active while List 2 is encoded. A forget instruction, in contrast, encourages participants to mentally separate the two lists. The shift in internal context that follows the forget instruction means that List 2 is encoded within a mental context that differs from the one associated with List 1. At test, the prevailing context therefore provides relatively effective retrieval cues for List 2 but poorer cues for List 1, resulting in impaired List 1 recall (Sahakyan and Kelley, 2002; for review, see Sahakyan et al., 2013).

Sahakyan and Kelley (2002) developed the *diversion paradigm* to test this contextual-change explanation of directed forgetting. Their approach was motivated in part by participants' reports about what they did after receiving an instruction to forget. Rather than somehow directly suppressing or erasing the preceding information, participants frequently reported intentionally distracting themselves by thinking about something unrelated to the experiment, often an autobiographical memory (Sahakyan and Kelley, 2002; Delaney et al., 2010). This observation suggested that a forget instruction might produce forgetting indirectly by encouraging participants to shift their thoughts away from the mental context in which List 1 had been encoded. Critically, explicitly inducing such a contextual shift between List 1 and List 2—without telling participants to forget—was sufficient to impair subsequent recall of List 1 (Sahakyan and Kelley, 2002; Sahakyan and Delaney, 2003). Diversionary tasks have included imagining one's childhood home and engaging in other forms of autobiographical or hypothetical thought designed to draw attention away from the current experimental context (Sahakyan and Kelley, 2002; Sahakyan and Delaney, 2003; Delaney et al., 2010).

The parallels between diversion-induced forgetting and traditional list-method directed forgetting are substantial. Like a forget instruction, engaging in diversionary thought between two lists reduces subsequent recall of List 1 relative to a control condition (Sahakyan and Kelley, 2002). The two manipulations also produce similar patterns of forgetting in children (Aslan and Bäuml, 2008), younger and older adults (Sahakyan et al., 2008), and interact with working memory capacity in similar ways (Delaney and Sahakyan, 2007). In particular, Delaney and Sahakyan (2007) found that individual differences in working memory capacity moderated memory performance following both directed forgetting and contextual-change manipulations, providing additional evidence that the two procedures engage overlapping processes. Moreover, both directed forgetting and diversion-induced context change require new learning following the forget cue or contextual shift for the forgetting effect to emerge (Pastötter and Bäuml, 2007), and both produce similar serial-position effects in subsequent recall (Sahakyan and Foster, 2009). Together, these similarities suggest that the amnesic consequences of a forget instruction can be reproduced simply by inducing a sufficiently strong change in mental context, even in the absence of an explicit instruction to forget.

Delaney et al. (2010) extended this work by proposing that context change is not an all-or-none phenomenon. Instead, the magnitude of forgetting should depend on the degree of discrepancy between the mental context associated with the original learning episode and the context induced by subsequent thought. Their reasoning follows from contextual theories of memory, according to which contextual features associated with an event serve as retrieval cues (Estes, 1955; Howard and Kahana, 2002; Mensink and Raaijmakers, 1988). As mental context changes over time and across experiences, the currently available cues become progressively less effective for retrieving information associated with an earlier context. Thus, a diversionary thought that transports a person psychologically farther from the present situation should produce a larger contextual shift and, consequently, greater forgetting than a thought that remains relatively similar to the current context.

Delaney et al. (2010) tested this hypothesis using autobiographical daydreaming tasks interpolated between two lists of words. In Experiment 1, participants studied List 1 and then either read a passage or imagined and drew the layout of their current home or their parents' home before studying List 2. Thinking about the parents' home produced significantly poorer List 1 recall than either the current-home or control condition, whereas thinking about the current home did not significantly impair recall relative to the control condition. Moreover, among participants who imagined their parents' home, the amount of time since they had last visited that home was negatively related to List 1 recall. Thus, the greater the temporal distance participants mentally traveled from the experimental context, the more pronounced the subsequent forgetting.

Experiment 2 provided an even stronger demonstration that the magnitude of a contextual shift matters. Participants again studied two lists separated by a diversion task, but this time they imagined a recent domestic or international vacation. Participants who thought about a domestic vacation recalled fewer List 1 words than control participants, whereas those who imagined an international vacation recalled fewer List 1 words than participants in either of the other conditions. Importantly, physical distance was also related to memory: across the two vacation conditions, greater distance between the experimental location and the imagined destination predicted poorer List 1 recall. Delaney et al. (2010) therefore concluded that the “mental travel” involved in daydreaming can disrupt access to recently encoded information and that the magnitude of this disruption increases as the imagined context becomes increasingly distinct from the current one.

This account is particularly relevant to the false fame effect because accurate fame judgments depend heavily on access to contextual information. To correctly reject a familiar but nonfamous name, participants must recollect that the familiarity associated with that name originated from its presentation on the earlier nonfamous list. If a shift in internal context reduces access to contextual details associated with that learning episode, participants may continue to experience familiarity when the name is encountered again but become less able to identify its source. Under these circumstances, familiarity can be misattributed to fame.

Buchli (2019) directly tested this possibility using a modified version of the Bjork and Bjork (2003) false fame paradigm. Rather than interpolating an instruction to remember or forget between the nonfamous and famous lists, participants either read from a textbook in the no-context change condition or completed the autobiographical diversion task used in previous research. Participants in the latter condition imagined their parents' home, mentally walked through its rooms, and drew its layout. This manipulation increased false fame judgments specifically for previously studied nonfamous names, consistent with the hypothesis that shifting internal context impaired recollection of the source of the names while leaving their familiarity intact. However, when participants in the daydreaming condition were prompted to recall the thoughts, feelings and emotions they experienced during the first phase of the experiment, thus reinstating the initial context in which the nonfamous names were presented, the false fame effect was eliminated.

## The present experiments

Taken together, these findings provide compelling evidence in favor of a contextual change account of the false fame effect. The aim of the current study is to replicate and extend these findings to provide further evidence in support of this account. If daydreaming tasks (Buchli, 2019; Delaney et al., 2010) reliably transport participants to another place and time, they should create a discrepancy between the context present during encoding of the nonfamous names and the context present during the subsequent fame judgment task. If this contextual discrepancy underlies the errors in recollection that produce increased false fame judgments, then thinking about a distant place and time should elicit more forgetting and greater susceptibility to false fame effects than thinking about a less distant place and time.

## Experiment 1

### Method

#### Participants

A total of 138 undergraduate students from Mercer University participated in Experiment 1 in exchange for course credit. Participants ranged in age from 18 to 22 years (M = 19.7); 57% identified as female and 43% as male. To be eligible to participate, individuals were required to be fluent English speakers and to reside in a university dormitory rather than with their parents.

The target sample size was determined *a priori* using G^*^Power (Faul et al., 2007) to conduct a power analysis for a factorial ANOVA. The effect size (η*p*^2^ = 0.360) was based on the context-change effect observed by Buchli (2019), with α set at.05 and statistical power set at.90 prior to data collection. This analysis yielded a target sample size of 138 participants.

#### Design

Three variables were manipulated in this experiment. The task interpolated between presentation of the nonfamous names and presentation of the famous names was manipulated between subjects, such that participants were randomly assigned to either the Draw Layout of Own Home condition (*near context change*), the Draw Layout of Parents' Home condition (*far context change*), or the Read Textbook Chapter Aloud condition (*control group; no context change*). Familiarity with (*old* vs. *new)* and Fame Status (*famous* vs. *nonfamous)* of the names presented during the fame judgment task were manipulated within subjects, such that participants were presented with both old and new names, some of which were famous and some of which were nonfamous.

#### Materials

The materials used in the present experiments were originally developed by Buchli (2019). As part of that research, a pilot study was conducted in which participants were shown a list of 300 famous names and asked to indicate whether each person was famous. From this pool, 36 names were selected that were correctly identified as famous 60%−70% of the time. The selected musicians, actors, artists, politicians, and other public figures were recognizable as famous, but not so well known that participants could readily recall the specific achievement for which they were famous. This criterion increased the likelihood that fame judgments would be based on familiarity rather than explicit recollection (Buchli, 2019; Jacoby et al., 1989). For each famous name, a nonfamous name was created that matched the famous name in gender, nationality, and the length of the first and last names. The same set of famous and nonfamous names was used in the present experiments. From these 72 names, two study lists were constructed: one comprised of 24 nonfamous names and one comprised of 24 famous names. A fame judgment list was also generated that consisted of 12 “old” famous names and 12 “old” nonfamous names taken directly from the study lists, as well as 12 “new” famous names and 12 “new” nonfamous names that participants had never seen before. Thus, 48 of the 72 names appeared in the lists that participants studied, and the remaining 24 appeared as “new” famous and nonfamous names during the fame judgment task. All of the study and fame judgment lists were counterbalanced, such that across participants, each of the famous and nonfamous names served as both an “old” name and a “new” name.

#### Procedure

Upon arriving for the experiment, participants were told that the investigator was interested in determining if there was a relationship between one's ability to remember names and one's ability to perform other sorts of cognitive operations. The experiment was conducted in individual testing rooms, and participants were seated in front of a computer next to the experimenter. Participants were informed that their first task would be to learn a list of nonfamous names 24 nonfamous names (e.g., Sebastian Weisdorf, Angelica Doyle) were subsequently presented, each name appearing on the computer screen for 5 s. Once all of names in the nonfamous list had been presented, participants in the control condition were asked to read a chapter aloud from an introductory psychology textbook for 90 s, so as to allow researchers to examine the potential relationship between reading speed and memory. Participants in the near context change condition were given the following instructions: *please close your eyes for a second and try to picture your current home. If you see it clearly, you may open your eyes. Now please describe your house from the moment you enter through the front door. Describe what you would see if you walked through every room including the details about the furniture and their location. Mentally walk through the house and draw a layout of all the rooms including the furniture. To save time, do not draw the pictures of furniture items, but instead use rectangles, squares, or circles to indicate a furniture item. Make sure you label them and explain the appropriate labels somewhere on your drawing. Please use the next 90 s to draw as much as possible*. Participants in the far context change condition were given the same instructions, except they were asked to imagine their parents' home (Delaney et al., 2010; Sahakyan and Kelley, 2002). Participants who currently lived with their parents, or who had never been to their parents' home were excluded from participating in the study. After the 90 s interval had expired, all participants were told that their next task would be to learn a list of famous names, but that they should keep trying to remember the list of nonfamous names. The list of 24 famous names (e.g., Minnie Pearl, Ray Ramano) was then presented, once again at a rate of 5 s each. After learning the famous names, participants completed a brief cued recall test on a subset of items from the famous-name list. This test was included to maintain consistency with the intentional learning instructions provided at the beginning of the experiment. For each item, participants were given a first name along with some of the letters of the last name (e.g., Christopher R_e_ _) and instructed to say the complete last name aloud to the experimenter. Names included on the cued recall test were excluded from the subsequent fame judgment task. After completing the memory test, participants were given 5 min to fill out a survey containing several questions about world geography, arts, and culture. This questionnaire served as a distractor task, and immediately preceded the fame judgment task. Once the questionnaire was finished, participants were told that their next task was to indicate, when a name appeared on the screen, whether that name referred to a famous person or a nonfamous person. If the name was famous, they were to say “yes,” if the name was not famous, they were to say “no.” Participants were further instructed that some of the names presented would be “old” names that appeared earlier in the experiment, while others would be entirely new names they had not seen before. Once the fame judgment task was completed, participants were debriefed and dismissed. A manipulation check was conducted to ensure that across participants, the number of miles traveled to their parents' home (for participants in the far context change condition*)* exceeded the number of miles traveled to their own home (for those in the near context change condition).

## Results

In Figure 1, the mean proportions of names judged to be famous in the No Context Change, Near Context Change, and Far Context Change conditions are displayed as a function of familiarity (old vs. new) and fame status (famous vs. nonfamous). First, the data was subject to a 3 (*interpolated task: no context change, near context change, far context change)* X 2 (*fame status: famous names, nonfamous names) X* 2 *(familiarity: old names, new names)* mixed ANOVA, revealing a significant three-way interaction, *F*(2, 135) = 22.13*, p* < 0.001, MSE = 0.011, η*p*^2^ = 0.247. Subsequently, in order to better assess the research hypothesis, separate mixed ANOVAS were conducted on nonfamous and famous names respectively.

**Figure 1:**
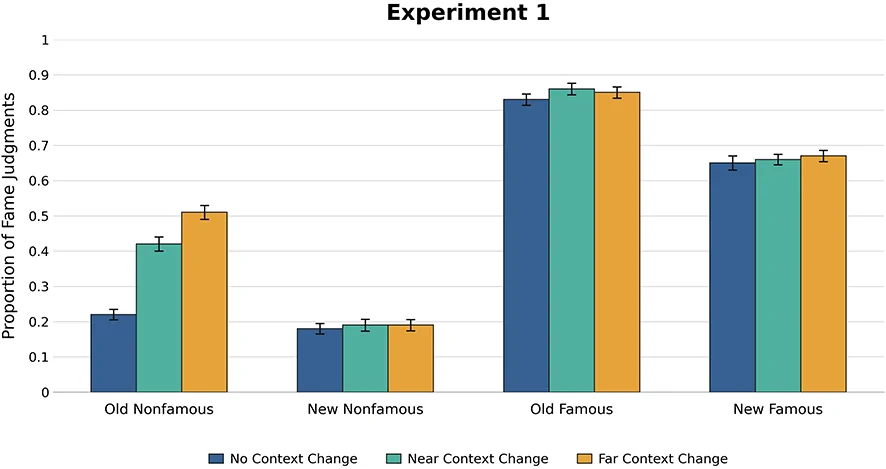
Proportions of famous and nonfamous names judged to be famous in Experiment 1 as a function of the intervening task that occurred between presentation of the nonfamous and famous names (Near vs. Far vs. No context change) and familiarity (Old Names vs. New Names). In the Near Context condition, participants drew the layout of their *own* home for 90 s. In the Far Context Change condition, participants drew the layout of their *parents'* home for 90 s. In the No Context Change condition, participants read an excerpt from an Introductory Psychology textbook for 90 s. Old names were familiar to participants, as they were presented previously in either the famous or nonfamous list. New names did not appear on either list, and were thus unfamiliar to participants. The error bars represent standard error of the mean.

### Nonfamous names

The mixed ANOVA conducted separately on the nonfamous names revealed a statistically significant interaction, *F*(1, 135) = 52.17*, p* < 0.001, MSE = 0.011, η*p*^2^ = 0.436, such that the observed differences in the proportion of fame judgments for the Near, Far, and No Context Change conditions were far greater for old nonfamous names than for new nonfamous names. That is, *post-hoc t*-tests^1^ confirmed that participants in the Far Context Change (*M* = 0.51, *SD* = 0.24) condition erroneously judged more nonfamous names to be famous than participants in the Near Context Change condition, (*M* = 0.42, *SD* = 0.24) *t*(90) = 3.47, *p* = 0.02, *d* = 0.48. Additionally, there was a higher proportion of false fame judgments in the Near Context Change (*M* = 0.42, *SD* = 0.24) condition than the No Context Change condition (*M* = 0.22, *SD* = 0.15), *t*(90) = 10.31, *p* < 0.001, *d* = 1.89. Analysis of the simple main effects confirmed that while these observed differences were significant for old nonfamous names, *F*(1, 135) = 76.057, *p* < 0.001, there were no significant differences for new nonfamous names, *F*(1, 135) = 0.10, *p* = 0.91 Thus, consistent with the context change hypothesis, participants who were prompted to draw the layout of their parents' home showed an increased susceptibility to false fame effects when compared to those who were prompted to draw the layout of their own homes or those who did not complete a task intended to shift their internal context.

To further assess whether the accuracy of participants' fame judgments was impaired due to prior shifts in their internal context, signal detection analyses were performed. The *d* prime scores for old and new names in each of the context change conditions are displayed in Table 1, along with proportions of hits and false alarms. A “hit” occurred when, during the fame judgment task, a participant gave a correct “yes” response to a famous name. A “false alarm” occurred when a participant gave an incorrect “yes” response to a nonfamous name. The resultant *d* prime score provides a quantitative index of how well a participant was able to distinguish between famous and nonfamous names. The higher a *d* prime score is, the better the respondent's discrimination ability. A mixed ANOVA was performed on the *d* prime scores, revealing a significant interaction, *F*(1, 135) = 10.76*, p* < 0.001, MSE = 0.27, η*p*^2^ = 0.138, indicating that the observed differences in discrimination ability across the Near, Far, and No Context Change conditions were far greater for old nonfamous names than new nonfamous names. That is, *post hoc t*-tests confirmed that participants' *d* prime scores were significantly lower in the Far Context Change (*M* = 1.01, *SD* = 0.28) condition than the Near Context Change condition (*M* = 1.36, *SD* = 0.29), *t*(90) = 2.74, *p* < 0.05, *d* = 0.75. Additionally, as predicted, the *d* prime scores in the Near Context Change condition (*M* = 1.36, *SD* = 0.29) were lower than the *d* prime scores in the No Context Change condition (*M* = 1.81, *SD* = 0.31), *t*(90) = 3.19, *p* = 0.009, *d* = 0.54. Analysis of the simple main effects confirmed that these observed differences were significant for old names, *F*(2, 135) = 20.005, *p* < 0.001, but not for new names, *F*(2, 135) = 2.18, *p* = 0.989.

**Table 1:** Proportion of hits, false alarms, and the associated *d*' score observed in Experiment 1 as a function of interpolated task (no context change vs. near context change vs. far context change), and familiarity (old vs. new).

Context change condition	Old names			New names		
	Hits	False alarms	*d*′	Hits	False alarms	*d*′
No context change	0.83	0.22	1.81	0.65	0.18	1.40
Near context change	0.86	0.42	1.36	0.66	0.19	1.39
Far context change	0.85	0.51	1.01	0.67	0.19	1.35

A “hit” occurred when participants correctly judged a famous name to be famous. A “false alarm” occurred when participants judged a nonfamous name to be famous. The *d*' provides a quantitative index of participants' ability to properly distinguish between famous and nonfamous names. Higher scores indicate better ability to discriminate. *d*′ = sensitivity index.

Thus, consistent with the context change hypothesis, participants were better able to discriminate between famous and nonfamous names in the No Context Change condition than either of the context change conditions, as indicated by the higher *d* prime score in the No Context Change condition.

### Famous names

The ANOVA revealed a significant main effect of familiarity, *F*(1, 135) = 266.15*, p* < 0.001, MSE = 0.008, η*p*^2^ = 0.663, indicating that collapsed across each of the three context change conditions, there was a higher proportion of fame judgments (*M* = 0.84, *SD* = 0.09) for old famous names than new famous names (*M* = 0.66, *SD* = 0.09). No other effects were significant.

## Mental miles and false fame

The distance, in miles, between Mercer University and each participant's current or parents' home was estimated. When a specific city was not provided, a representative city in the state indicated by the participant was used. The Near Context Change group traveled significantly fewer miles (*M* = 2.5 miles, SD = 2.1 miles) than the Far Context Change group (*M* = 165 miles, SD = 210 miles), *t*(90) = 5.25, *p* < 0.001, *d* = 1.10. For the combined Near and Far Context Change conditions, the correlation between distance in miles and the proportion of old nonfamous names judged to be famous was significant, *r*(90) = 0.29, *p* = 0.005. Therefore, thinking about a more geographically distant location was associated with greater susceptibility to the false fame effect.

## Discussion

According to the context change hypothesis, false fame effects emerge when the contextual cues that were present during encoding of the nonfamous names are no longer present during the fame judgment task. Hence, daydreaming tasks that introduce a significant degree of discrepancy between the initial context in which the nonfamous names are learned and the context in which fame judgments are made should result in the highest proportion of false fame judgments. Consistent with this hypothesis, the more “mental miles” participants traveled during the daydreaming task, the greater the resultant false fame effect. That is, participants who imagined and described the layout of their parents' homes later judged more nonfamous names to be famous than participants who imagined the layout of their own home. Signal detection analyses also indicated that participants who were prompted to think about their own home or their parents' home were less able to accurately distinguish between famous and nonfamous names than participants who did not shift their internal context. The aim of Experiment 2 was to replicate and extend these findings by determining if thinking about an international vacation after encoding the nonfamous names would result in a higher proportion of false fame judgments than thinking about a domestic vacation (Delaney et al., 2010).

## Experiment 2

### Method

#### Participants

A total of 138 undergraduate students from Mercer University participated in Experiment 2 in exchange for course credit. Participants ranged in age from 18 to 24 years (M = 20.2); 62% identified as female and 38% as male. To be eligible to participate, individuals were required to be fluent English speakers and to have traveled both outside the state of Georgia and internationally within the previous 5 years.

The sample size for Experiment 2 was established *a priori* using G^*^Power (Faul et al., 2007) to conduct a power analysis for a factorial ANOVA. The analysis used the context-change effect reported by Buchli (2019) (η*p*^2^ = 0.360), with α = 0.05 and statistical power set at 0.90 prior to data collection. Based on these parameters, the required sample size was 138 participants.

#### Design, materials, and procedure

The materials, design, and procedure of Experiment 2 were identical to those of Experiment 1, except that in the near context change condition, instead of drawing the layout of their current home, participants were prompted to describe a recent vacation that took place within the USA but outside of Georgia. Instead of drawing the layout of their parents' home, participants in the far context change condition were prompted to describe a recent international vacation (one that took place outside of the USA). All vacations must have taken place within the last 5 years. Participants were given 90 s to describe their vacation, and were asked to indicate what they “saw, felt, smelled and experienced.” Like the drawing task, prompting participants to invoke such memories has reliably been demonstrated to shift one's internal context, as it encourages participants to mentally transport themselves to the time and place in which the vacation occurred. This task effectively mimics the cognitive processes that are engaged during daydreaming or mind wandering, thus causing considerable fluctuation in the contextual cues that are available and impeding successful recollection of previously learned information (Delaney et al., 2010; Sahakyan and Kelley, 2002). Participants in the no context change condition were again prompted to read aloud from an introductory psychology textbook for 90 s instead of completing the vacation description task. All participants selected for the experiment had vacationed domestically and internationally within the last 5 years. They were randomly assigned to either the Far, Near, or No Context Change condition. A manipulation check was conducted to ensure that across participants, the number of miles traveled internationally (for those in the far context change condition*)* was greater than the number of miles traveled domestically (for those in the near context change condition).

## Results

In Figure 2, the mean proportions of names judged to be famous in each of the context change conditions are displayed as a function of familiarity (old vs. new) and fame status (famous vs. nonfamous). To determine whether inducing a change in mental context affects subsequent judgments of fame, once again a three (*no context change vs. near context change vs. far context change)* X 2 *(famous names vs. nonfamous names)* X 2 (*old names vs. new names)* mixed ANOVA was analyzed, followed by separate mixed ANOVAs on the nonfamous and famous names respectively. The initial ANOVA again revealed a significant three-way interaction, *F*(2, 135) = 23.50*, p* < 0.001, MSE = 0.013, η*p*^2^ = 0.258.

**Figure 2:**
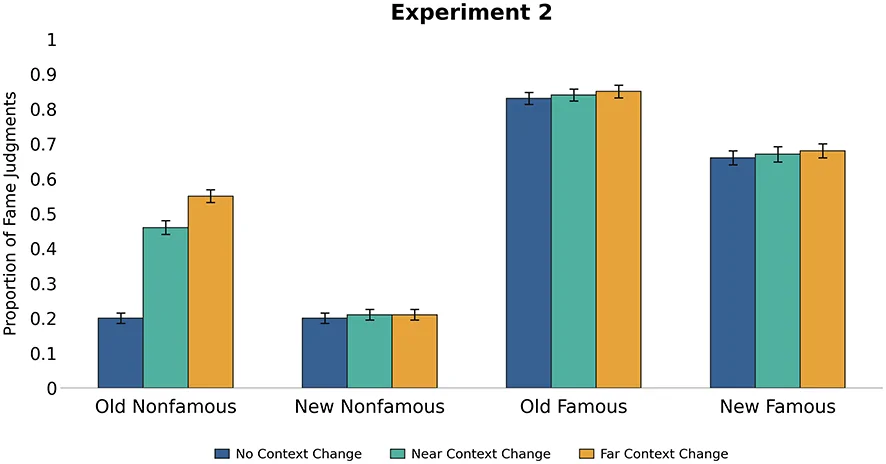
Proportions of famous and nonfamous names judged to be famous in Experiment 2 as a function of the task interpolated between presentation of the nonfamous and famous names (Near vs. Far vs. No context change) and familiarity (Old Names vs. New Names). In the Near Context condition, participants spent 90 s describing a recent vacation within the USA, but outside the state of Georgia. In the Far Context Change condition, participants described a recent vacation outside of the USA for 90 s. In the No Context Change condition, participants read a textbook chapter aloud for 90 s. Old names were those presented previously in the experiment, while new names had not been studied by participants. The error bars indicate standard error of the mean.

### Nonfamous names

Again, a significant interaction emerged, *F*(1, 135) = 47.32 *p* < 0.001, MSE = 0.012, η*p*^2^ = 0.412, such that the observed differences in the proportion of fame judgments for the Near, Far, and No Context Change conditions were far greater for old nonfamous names than for new nonfamous names. *Post hoc t*-tests again confirmed that there was a higher proportion of false judgments of fame in the Far Context Change (*M* = 0.55, *SD* = 0.21) condition than the Near Context Change condition (*M* = 0.46, *SD* = 0.27), *t*(90) = 3.48, *p* = 0.03, *d* = 0.49. There was also a higher proportion of false judgments of fame in the Near Context Change condition (*M* = 0.46, *SD* = 0.27) than the No Context Change condition (*M* = 0.20, *SD* = 0.15), *t*(90) = 9.97, *p* < 0.001, *d* = 1.65. Analysis of the simple main effects confirmed that while these observed differences were significant for old nonfamous names, *F*(2, 135) = 74.544, *p* < 0.001, there were no significant differences between the context change conditions for new nonfamous names *F*(2, 135) = 3.64, *p* = 0.824. Thus, consistent with the context change hypothesis, thinking about a vacation outside of the USA increased participants' susceptibility to the false fame effect more than thinking about a vacation within the USA.

Once again, in an attempt to find converging evidence that participants' ability to distinguish between famous and nonfamous names was negatively impacted by prior shifts in internal context, *d* prime scores were calculated for both old and new names in each of the context change conditions and subject to a mixed ANOVA (see Table 2). Such analysis revealed a significant interaction, *F*(2, 135) = 9.982*, p* < 0.01, MSE = 0.571, η*p*^2^ = 0.131, indicating that the observed differences in discrimination ability across the Near, Far, and No Context Change conditions were far greater for old nonfamous names than new nonfamous names. *Post hoc t*-tests again confirmed that participants' *d* prime scores were significantly lower in the Far Context Change condition (*M* = 1.16, *SD* = 0.27) than the Near Context Change condition (*M* = 1.63, *SD* = 0.24), *t*(90) = 2.80, *p* < 0.01, *d* = 0.62. *D* prime scores were also lower in the Near Context Change condition (*M* = 1.16, *SD* = 0.27) than the No Context Change condition (*M* = 2.20, *SD* = 0.29), *t*(90) = 3.49, *p* < 0.01, *d* = 0.54. Analysis of the simple main effects confirmed that these observed differences were significant for old names, *F*(2, 135) = 12.886, *p* < 0.001, but not for new names, *F*(2, 135) = 7.29, *p* = 0.43.

**Table 2:** Proportion of hits, false alarms, and the associated *d*' score observed in Experiment 2 as a function of interpolated task (no context change vs. near context change vs. far context change), and familiarity (old vs. new).

Context change condition	Old names			New names		
	Hits	False alarms	*d*′	Hits	False alarms	*d*′
No context change	0.83	0.23	2.20	0.66	0.20	1.32
Near context change	0.84	0.46	1.63	0.67	0.21	1.35
Far context change	0.85	0.55	1.16	0.68	0.21	1.36

A “hit” indicates a correct identification of a famous name as famous, while a false alarm indicates an incorrect labeling of a nonfamous name as famous. *D*' scores provide quantitative measure of how well participants were able to properly distinguish between famous and nonfamous names; higher scores indicate superior discrimination ability. *d*′ = sensitivity index.

Therefore, once again, as predicted by the context change hypothesis, participants' ability to discriminate between famous and nonfamous names was significantly impeded by a prior shift in internal context.

### Famous names

A significant main effect of familiarity emerged, *F*(1,135) = 122.40*, p* < 0.001, MSE = 0.018, η*p*^2^ = 0.476 such that collapsed across each of the three context change conditions, participants correctly deemed a higher proportion of old famous names (*M* = 0.84, *SD* = 0.12) to be famous than new famous names (*M* = 0.67, *SD* = 0.14). No other effects were significant.

### Mental miles as a predictor of false fame

The distance, in miles, between Mercer University and each participant's vacation destination was estimated. When a specific city was not provided, a popular tourist or study-abroad destination in the state or country indicated was used. The Near Context Change group traveled significantly fewer miles (*M* = 893 miles, *SD* = 717 miles) than the Far Context Change group (*M* = 4,206 miles, *SD* = 2,550 miles), *t*(90) = 8.48, *p* < 0.001, *d* = 1.77. For the combined Near and Far Context Change conditions, the correlation between distance in miles and the proportion of old nonfamous names judged to be famous was significant, *r*(90) = 0.32, *p* = 0.002. Therefore, thinking about travel to a farther destination was associated with greater susceptibility to the false fame effect.

## Discussion

Once again, the number of “mental miles” traveled during the daydreaming task determined the magnitude of the resultant false fame effect. Participants who described an international vacation made more false judgments of fame than participants who described a domestic vacation, and participants who described a domestic vacation made more false fame judgments than participants in the control condition. Signal detection indices also confirmed that participants prompted to describe either type of vacation had much more difficulty distinguishing between famous and nonfamous names than participants who did not shift their internal context.

### General discussion

The false fame effect occurs when participants erroneously deem previously presented nonfamous names to be famous as a result of impaired recollection of the earlier learning episode, but an enduring sense of familiarity with the names. According to the contextual change account of false fame effects, more false judgments of fame should occur in situations where participants are no longer able to access the contextual cues that were present during encoding of the nonfamous names when completing the fame judgment task. For instance, when a context change is introduced, either as a result of the passage of time (Jacoby et al., 1989) or a forget instruction (Bjork and Bjork, 2003), fewer contextual details that were present during initial learning are available during the fame judgment task, and successful recollection of these details is impaired. Participants' familiarity with the names, however, unaffected by these shifts in internal context, causes them to incorrectly conclude that the nonfamous names are famous. Relatedly, prompting participants to daydream and thus mentally transport themselves to another location and point in time after encoding the nonfamous names should result in impaired recollection and a greater susceptibility to false fame than tasks that do not incorporate such mental time travel, as these tasks reliably shift participants' internal context (Buchli, 2019; Delaney et al., 2010; Sahakyan and Kelley, 2002). Furthermore, daydreaming tasks that introduce more discrepancy between the initial context in which the nonfamous names are learned and the subsequent context in which the names are judged should elicit more false fame judgments than tasks that introduce less discrepancy. One measure of such discrepancy is the number of “mental miles” participants must travel when engaged in the daydreaming task. Thinking of one's parents' home or an international vacation, for example, should require one to mentally travel a greater distance than thinking about one's own home or a domestic vacation, though either manipulation would be expected to shift participants' internal context. Hence, according to the contextual change account, a *far* context change should elicit more false judgments of fame than a *near* context change, and a *near* change should result in more false fame judgments than tasks that are not expected to shift participants' internal context.

As predicted, across two experiments, inducing a far context change, either by instructing participants to draw the layout of their parents' home or to describe a vacation outside of the USA, resulted in more false fame judgments than inducing a near context change, either by instructing participants to draw the layout of their own home or to describe a domestic vacation. Similarly, eliciting a near context change resulted in more false fame judgments than tasks that failed to shift participants' internal context. Importantly, the mental-miles analyses provided converging evidence for this pattern. Across the near and far context-change conditions, the geographic distance participants mentally traveled was positively associated with the proportion of old nonfamous names subsequently judged to be famous in both experiments. Thus, susceptibility to false fame increased not only as a function of the experimentally defined context-change conditions, but also as a function of the magnitude of the mental distance traveled during the context-change task. Collectively, these results provide more compelling evidence in favor of the contextual change account of false fame effects.

The present findings also converge with the directed-forgetting literature reviewed earlier. As discussed in the Introduction, the contextual change account of list-method directed forgetting proposes that a forget instruction encourages participants to shift their internal mental context, thereby reducing the accessibility of information encoded prior to the shift (Sahakyan and Kelley, 2002). Importantly, directed forgetting can be reproduced without an explicit instruction to forget by inserting diversionary thought between learning episodes, particularly when that thought encourages participants to mentally transport themselves to another time or place (Delaney et al., 2010). The present findings extend this work beyond intentional forgetting to memory distortion. Rather than simply making previously encoded information less accessible, changes in internal context appear capable of altering how residual familiarity is interpreted: when recollection of the original learning context is impaired, familiarity with previously encountered nonfamous names may instead be misattributed to fame. The mental-miles findings are particularly consistent with this account because greater mental distance was associated with greater memory distortion, paralleling previous evidence that greater mental distance is associated with greater forgetting (Delaney et al., 2010).

While the results reported here are interesting and consistent with almost 50 years' worth of research on context-dependent memory (Godden and Baddeley, 1975; Smith, 1979, 1994; Smith et al., 1978; Smith and Vela, 2001), many important questions remain. For example, it has been established that impaired recollection combines with persistent feelings of familiarity to cause participants to make more false judgments of fame after a mental context shift. However, such errors in recollection could manifest in a number of ways. One possibility is that when presented with a familiar name during the fame judgment task, participants recognize the name, but incorrectly assume that the name was presented as part of the famous list. This error derives from faulty source memory or an inability to properly distinguish between the famous and nonfamous lists. Another possibility is that, when presented with a familiar nonfamous name, participants do not recognize it as a name that was presented earlier, but on the basis of the familiarity that they experience, decide that it must be a new famous name. This error reflects a much more general, pervasive kind of episodic forgetting. Further research should be conducted to determine which errors are more likely to persist after shifts in internal context, and how contextual reinstatement reduces such errors (Bjork and Bjork, 2003; Buchli, 2019).

An additional question concerns precisely what constitutes the contextual change produced by the autobiographical tasks used here. Although geographic distance provides one readily quantifiable dimension of contextual discrepancy, the near and far conditions likely differed along several dimensions simultaneously. For example, one's parents' home may differ from one's current home not only in physical location, but also in the amount of time since the location was last visited, the period of one's life associated with it, its personal relevance, and the autobiographical memories and emotions it evokes. Similarly, domestic and international vacations may differ in geographic distance, recency, novelty, cultural distinctiveness, emotional salience, and the richness of the memories they elicit. Consequently, the present results should not be interpreted as demonstrating that physical distance itself is solely responsible for the observed context-change effects. Rather, mental miles may serve as one measurable indicator of a broader shift in internal context.

Future research could therefore attempt to isolate these dimensions experimentally. For example, geographic distance could be manipulated while holding recency approximately constant, or participants could imagine two locations that are similarly distant but differ in familiarity or personal relevance. Conversely, temporal distance could be manipulated while geographic location remains constant by asking participants to imagine themselves in the same location at different points in their lives. Such designs would help determine whether spatial distance, temporal distance, autobiographical relevance, novelty, or some combination of these factors is most important for producing changes in false fame. They would also help establish whether “mental miles” have a unique relationship with false fame after other dimensions of contextual discrepancy are controlled.

It is also important to recognize that context change is unlikely to be an all-or-none phenomenon. Even the control task used in the present experiments may have produced some degree of internal context change because participants transitioned from studying names to reading material aloud, thereby changing their cognitive activity and attentional state. The critical distinction, therefore, may not be between the presence and absence of context change, but rather the *magnitude* of the change. This interpretation is consistent with the graded pattern observed across the control, near, and far conditions, as well as the positive relationship between mental miles and false fame judgments. Future studies could test this possibility more directly by including several comparison tasks designed to produce progressively larger shifts in cognitive, spatial, temporal, or autobiographical context. Such an approach would permit a more precise characterization of the relationship between the magnitude of contextual change and susceptibility to false fame.

Future research should also explore whether manipulating the environmental context in which nonfamous names are learned and subsequently judged as famous or nonfamous influences the proportion of false fame judgments observed. The contextual change account would predict that making fame judgments in the same physical environment where the nonfamous names were learned should result in fewer false judgments of fame than if the physical environment differs between initial learning and the fame judgment task. Similarly, manipulating participants' mood or other aspects of their internal state to determine whether false fame effects are mood-congruent or state dependent would provide valuable insights as to how robust or generalizable the findings reported here are to related phenomena (Eich, 1989; Eich and Metcalfe, 1989; Grant et al., 1998; Ucros, 1989).

Another potential question to consider relates to individual differences in one's susceptibility to the false fame effect. Prior research, for example, suggests that personality traits like absorption (Clancy et al., 2002) and fantasy-proneness (Spanos et al., 1991) are more common in individuals who demonstrate higher recollection of false memories. Absorption refers to a dispositional tendency to become extremely engrossed in one's mental imagery and daydreams. Similarly, fantasy-prone individuals regularly devote a significant amount of time to imaginative activities. Horselenberg et al. (2006) found, however, that neither fantasy proneness nor absorption predicted the proportion of false fame judgments observed. These results contradict what would be predicted by the contextual change account, as those who frequently engage in fantasy and vivid daydreaming would be expected to show the greatest propensity to experience internal shifts in context. Interestingly, however, Peters et al. (2007) found that individuals who claimed to have highly detailed, vivid memories of episodes from a past life after hypnosis with a reincarnation therapist showed a higher proportion of false fame judgments than individuals without such implausible recollections. This provides some evidence that individuals prone to suggestion imposed by guided imaginative daydreaming under hypnosis have more difficulty differentiating between famous and nonfamous names. Future research should examine these discrepancies to see when differences in one's capacity to engage in daydreaming predict and do not predict vulnerability to false memories.

## Data Availability

The raw data supporting the conclusions of this article will be made available by the authors, without undue reservation.

## Footnotes

1 All *post hoc* tests reported were Bonferroni corrected for multiple comparisons.
